# Rapidly (and Successfully) Translating Novel Brain Radiotracers From Animal Research Into Clinical Use

**DOI:** 10.3389/fnins.2020.00871

**Published:** 2020-10-01

**Authors:** Robert C. Shaw, Gilles D. Tamagnan, Adriana Alexandre S. Tavares

**Affiliations:** ^1^BHF Centre for Cardiovascular Sciences, University of Edinburgh, Edinburgh, United Kingdom; ^2^Edinburgh Imaging, University of Edinburgh, Edinburgh, United Kingdom; ^3^XingImaging, LLC, New Haven, CT, United States

**Keywords:** novel radiotracer, translational, brain, animal research, development and discovery

## Abstract

The advent of preclinical research scanners for *in vivo* imaging of small animals has added confidence into the multi-step decision-making process of radiotracer discovery and development. Furthermore, it has expanded the utility of imaging techniques available to dissect clinical questions, fostering a cyclic interaction between the clinical and the preclinical worlds. Significant efforts from medicinal chemistry have also made available several high-affinity and selective compounds amenable for radiolabeling, that target different receptors, transporters and enzymes *in vivo*. This substantially increased the range of applications of molecular imaging using positron emission tomography (PET) or single photon emission computed tomography (SPECT). However, the process of developing novel radiotracers for *in vivo* imaging of the human brain is a multi-step process that has several inherent pitfalls and technical difficulties, which often hampers the successful translation of novel imaging agents from preclinical research into clinical use. In this paper, the process of radiotracer development and its relevance in brain research is discussed; as well as, its pitfalls, technical challenges and future promises. Examples of successful and unsuccessful translation of brain radiotracers will be presented.

## Introduction

Single photon emission computed tomography (SPECT) and positron emission tomography (PET) rely on the *in vivo* detection and quantification of the radiotracer distribution and binding to a specific biological target in the living body (Salvadori, 2008). These techniques are at the leading edge of molecular imaging and allow for exceptional target specificity and high sensitivity (Haberkorn and Eisenhurt, 2005; Salvadori, 2008; Kemp et al., 2010). PET and SPECT imaging is based on the radiotracer principle, which states that the radiotracer does not alter or perturb the biological system under investigation. This is only possible when the injected mass of a radiotracer occupies a small percentage of the target, i.e., the microdosing principle (Ruth, 2009). Radiotracer imaging allows for studies investigating neurotransmission, metabolism, regional cerebral blood flow and pharmacology *in vivo*. Consequently, PET and SPECT imaging can assist in diagnosing multiple neurodegenerative and neuropsychiatric disorders. They also provide imaging biomarkers to track disease development and monitor the effects of drugs on disease progression. Finally, radionuclide molecular imaging techniques can be used to determine optimal dosing for new drugs via microdosing experimental set-up and can aid with accelerating the implementation of personalized medicine (Brooks, 2005; Agdeppa and Spiker, 2009; Van de Bittner et al., 2014).

Despite the tremendous potential of PET and SPECT imaging, only a limited number of central nervous system (CNS) targets are currently used in humans; yet there are thousands of potential brain proteins not yet explored. This limited availability may in part be explained by the wide range of ever-growing, strict, criteria that must be fulfilled prior to radiotracer regulatory approval and by the empirical nature of radiotracer discovery (Brust et al., 2014; Van de Bittner et al., 2014). In this review, different aspects associated with brain radiotracer discovery and development will be discussed. Suggestions of alternative approaches to improve the flow through the pipeline will be also provided, alongside examples of radiotracers successful and unsuccessful in their translation to human research.

## The Process of Radiotracer Discovery and Development

**FIGURE 1:**
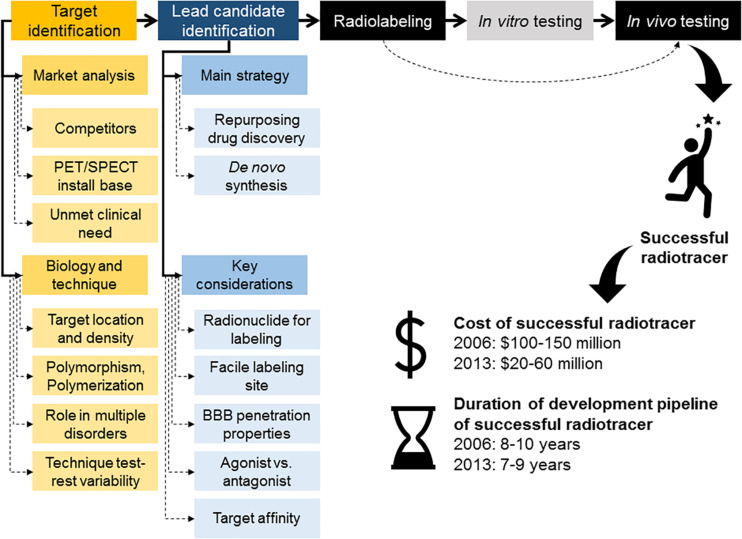
Overview of radiotracer discovery and development pipeline. Main steps in the radiotracer discovery and development pipeline, including typical development costs and timelines for development of a successful radiotracer. A strategy to accelerate the process of radiotracer development involves removal of the *in vitro* testing of the radiolabeled compound and instead progress with the radiolabeled compound straight into *in vivo* testing.

Despite the similarities between drug and radiotracer discovery and development processes, imaging agent development has the flexibility to aim at investigating functional and non-functional targets, as long as they play a role on a given disorder or mechanism of interest and meet imageability requirements (Agdeppa and Spiker, 2009). Unfortunately, the relatively small market sizes for radiotracer commercialization compared with the drug market, together with radiolabeling constrains and regulatory/intellectual property challenges, represent restrictions to the innovation potential in radiotracer discovery and development. In fact, based on 2004 estimates, the total imaging market was only 1% of the total therapeutic market (Nunn, 2006a). This demonstrates that the majority of the radiotracers developed will fall into a small/specialty market size. Furthermore, in 2006, it was estimated that a diagnostic imaging agent takes ~8–10 years to develop at a cost of between $100 and $150 million with a potential return in sales of only $200–400 million per year, even for highly used diagnostic agents, such as the nuclear cardiology radiotracer Cardiolite^TM^, Lantheus. This value was gained through associated costs from therapeutic drug development as there were not publicly available data at this time on costs for developing imaging agents (Nunn, 2006b). Conversely, costs to develop a novel therapeutic drug are around $850 million over 14 years with a potential return of up to as much as $3.4 billion (Nunn, 2006a, b). Later, in Zimmermann (2013) estimated the cost of a conventional drug development at €82–300 million over 10–15 years; while the development of novel radiotracers for diagnostic purposes was calculated to be €20–60 million over 7–9 years (Figure 1). However, this is a conservative measure looking at money invested in a successful candidate not taking into account the investment in failed candidates or candidates that did not make it to completion. If the cost of investment in research into unsuccessful targets was included this figure could be as much as 300% higher (Zimmermann, 2013). In March, Wouters et al. (2020) reported that when accounting for the costs of failed drug trials (between 2009 and 2018), the mean investment was $1,335.9 million. Analysis of the success rate of new drugs per stage of development, excluding regulatory approval, shows that the lowest success is achieved at the preclinical stages with only ∼30% success rate. This is lower than first in human success rate that averages at ~75% (Takebe et al., 2018). It is possible that the drop in development costs of successful drugs and radiotracers relates with improvement of preclinical methods, which in the case of PET/SPECT imaging might stem from the development of dedicated preclinical imaging platforms. Notwithstanding, the overall cost (i.e., corrected for failed candidates) has not reduced with time. From these data, it is clear that the commercialization of diagnostic imaging agents will not generate the same return as pharmaceutical drugs. Thus, partnerships between academia, the pharmaceutical industry and imaging companies improves the viability of radiotracer discovery and development in light of the increasing demand for personalized medicine. Over the years, the academic community has been invaluable to explore new high risk/high gain areas; demonstrating the feasibility of new imaging approaches. Unsurprisingly, academia has been credited for the majority of PET and SPECT radiotracers discovery to date. In turn, industry can facilitate the route to commercialization for radiotracers discovered in academia (Agdeppa and Spiker, 2009). Furthermore, partnering between pharmaceutical companies, imaging companies and academia can allow for the conversion of failed therapeutic agents into radiotracers. Abandoned drug candidates and families of compounds, which failed due to toxicity or short biological half-life, can sometimes be repurposed as PET or SPECT radiotracers. This is due to the radiotracer principle allowing for the safe use of very low (picomolar) concentrations of compounds that are toxic at higher concentrations. Furthermore, the rapid clearance of a given compound, which is suboptimal for drug candidates, may be suitable and even desirable, for a PET or SPECT radiotracer (Willmann et al., 2008).

The process of radiotracer discovery and development has been described in various ways, however, one aspect of this process is consistent across the field: developing radiotracers is a multidisciplinary iterative process. Each stage of the process can feedback to a prior stage and/or input the subsequent stage (Salvadori, 2008; Agdeppa and Spiker, 2009; Barth and Need, 2014; Van de Bittner et al., 2014). Below we provide an overview of the key stages in the process of radiotracer discovery and development.

### Evaluation of Market Opportunities and Prioritization of Research Questions to Target

At early stages of radiotracer discovery, multiple factors must be considered beyond the scientific challenges alone (Figure 1). It is important to understand from the outset how the radiotracer would be used and then established predefined performance criteria required for success. For example, one could ask whether the development of a novel radiotracer for a given target would enhance the ability to image such target by improving target:non-target ratios, reducing off target binding or kinetics *in vivo*. For previously unexplored targets, it would be important to provide convincing evidence that the novel radiotracer will specifically label the target or mechanism that they are designed to measure (Agdeppa and Spiker, 2009).

Given the expensive and time-consuming nature of radiotracer discovery and development, which ultimately culminates with a small/specialty market, it is also important to perform a “market search” at the inception of radiotracer discovery. Obtaining insights from physicians can be useful to understand the need for a given imaging tool in clinical practice or research. In short, if imaging with a radiotracer for an unmet clinical need would not be “prescribed,” then the development of such radiotracer may be unnecessary. In the basic research context, which accounts for the majority of these PET and SPECT imaging, the utility of a radiotracer can be difficult to anticipate. Potential use can be appraised by discussing this among colleagues, preclinical scientists and experts from pharmaceutical companies, in order to determine preclinical use and human translational potential of a given radiotracer (Van de Bittner et al., 2014). Commercialization potential is important when selecting projects and in practice; technically feasible projects may not be a priority due to a limited potential return on investment (Nunn, 2006a). A careful evaluation of the market size may also be driven by current/future install bases in clinical use and modality constrains. For example, SPECT equipment is more widely distributed and numerous in comparison with PET (around 12 530 multi-head SPECT scanners vs. around 1000 PET scanners in the United States of America in 2008) (Agdeppa and Spiker, 2009). Thus, the development of a novel SPECT radiotracer might be preferred over a PET radiotracer if the SPECT imaging performance is adequate. This is especially true as the physical limits of PET and SPECT detection are approached in the clinical setting as they have been approached in the preclinical setting (Mariani et al., 2008; Seo et al., 2008; Ruth, 2009). For example, in the preclinical setting, small animal SPECT scanners have higher resolution than PET scanners, although this comes at the expense of relatively lower sensitivity (de Kemp et al., 2010; Koba et al., 2013). PET is arguably superior to SPECT in regards to being able to directly measure the attenuation effect of the object being viewed; as well as having a higher resolution and accuracy in quantitative assessment of regional concentrations of a radiotracer (Alavi and Basu, 2008; Ruth, 2009). The higher sensitivity gives PET the ability to measure targets with lower expression. However, PET does still have limitations and challenges. Short-lived PET radionuclides, such as ^15^O and ^11^C, require a cyclotron in close proximity to the PET scanner. This may limit widespread market distribution, but it can be advantageous during early stages of radiotracer discovery and development as it will be detailed below. PET scanning also incurs higher costs in comparison with SPECT scanning (Ottobrini et al., 2006; von Schulthess and Burger, 2010). Targeting specific molecules so that they can be easily labeled for both PET and SPECT imaging should be considered in the early stages of radiotracer design in order to reach users in both modalities. This keeps options open and can save time and money in long term development.

From a commercialization point of view, similarly to therapeutic drugs, knowing the intellectual property (IP) landscape before project conception can help identify opportunities and competition. It is important to be up to date on the status of technology, other applicable imaging agents and non-imaging biomarkers, regardless of the imaging modality. For example, magnetic resonance imaging (MRI) research is improving endogenous signal without the use of contrast agents. Blood biomarkers and non-imaging biomarkers are other forms of competition and could limit the use of novel imaging agent, although these diagnostics lack spatial information that can be obtained with imaging. If a cheap blood test can give the same information as an expensive time consuming scan then it is unlikely that it will be adopted in the clinical setting. These aspects are important to consider, especially due to the long times associated with the development of novel radiotracers. Moreover, consideration of the impact of a novel radiotracer on health economics, in light of the reimbursement issues, may be valuable when aiming at commercialization of a novel radiotracer (Agdeppa and Spiker, 2009). Alternative approaches, less focused on the commercialization of the novel radiotracer, have suggested to start the brain radiotracer development program based on assessing an unmet medical need or based on an expectation in human disease imaging (Van de Bittner et al., 2014).

In summary, when initiating the journey of radiotracer discovery and development, the researcher must look beyond purely the scientific scope and should address important questions. If the purpose of that radiotracer development is to attract investment and lead to commercialization: (1) is there a market or a medical need that justifies the development of this radiotracer?; (2) will the quality of the science and technology behind the new molecule be attractive to an investor?; (3) is it feasible and will it comply with regulations?; and (4) in this limited competitive landscape, will the novel radiotracer stand a chance? An example of a recently developed successful radiotracer with potential to have wider impact in the field of brain research and clinical imaging is ^18^F-MNI1126, a radiotracer targeting the synaptic vesicle glycoprotein 2A (SV2A) (Mercier et al., 2017; Constantinescu et al., 2019).

### Target Identification

Traditionally, therapeutic drug targets have an important mechanistic effect in disease processes, such as, inhibiting the target may alter/reduce disease severity or progression (Pritchard et al., 2003). An important difference between therapeutic drugs and diagnostic radiotracers’ target identification step is that the latter can be developed for targets that play an important role in a given disease, yet have no functional effect in modifying disease processes. A pivotal example of these so called “non-functional” targets is amyloid imaging, where structural proteins are targeted (Klunk et al., 2004). This means that a radiotracer target needs only have altered expression, occupancy or function in a given disorder, resulting in a wide range of potential targets for *in vivo* imaging, which in turn will allow for a given radiotracer to be applied to investigate several different medical or biological questions (Agdeppa and Spiker, 2009; Van de Bittner et al., 2014).

To assist with the target identification during radiotracer discovery, it is also important to consider the location and amount of target assessable for high quality *in vivo* imaging. An essential property for *in vivo* imaging using radiotracers is the concept of binding potential (*BP*), which provide a measurement of radiotracer-target interaction as a function of the total target density (*B*_*max*_) and the radiotracer binding affinity (*K*_*D*_). The suggested ideal *BP* values for *in vivo* imaging in the literature can vary considerably from 1.5 (Barth and Need, 2014) up to 10 (Patel and Gibson, 2008). Some have proposed, as a rule of thumb, that a *BP* of 5 is a suitable value for quantitative PET imaging, especially in the clinical setting where kinetic modeling techniques are not always available (Van de Bittner et al., 2014). When assessing which target to tackle during radiotracer discovery, it is possible to estimate the ideal *K*_*D*_ value for the prospective candidate radiotracer from the known *B*_*max*_. When the *B*_*max*_ is unknown, it can be measured using autoradiography, binding assays or quantitative immunohistochemistry methods. *In vivo, BP* can be expressed in three different forms depending on the reference used, *BP*_*ND*_, *BP*_*F*_, and *BP*_*P*_. *BP*_*ND*_ refers to the ratio of radioligand specifically bound to its target vs the amount binding non-specifically (the non-displaceable amount). *BP*_*F*_ refers to the ratio of specifically bound radiotracer to free fraction and *BP*_*P*_ refers to the total concentration in the plasma that is available (excluding ligand irreversibly bound to plasma proteins) (Innis et al., 2007). Examples of brain SPECT and PET radiotracers previously developed and correspondent *BP*_*ND*_ values are presented in Table 1 below.

**TABLE 1:** Examples of brain SPECT and PET radiotracers previously developed and associated *BP*_*ND*_ values in healthy humans.

Radiotracer	Target (high to low density)	*BP*_*ND*_	References
^123^I-Iomazenil	GABA receptors	12.50 ± 0.05 (*n* = 10)	Abi-Dargham et al., 1994
^123^I-(R,R)l-QNB	mACh receptors	4.85 (*n* = 11)	Norbury et al., 2004
^123^I-5-IA85380	α4β2-nACh receptors	4.43 ± 0.06 (*n* = 6)	Fujita et al., 2003
^123^I-β-CIT	Dopamine transporters	6.66 ± 1.54 (*n* = 5)	Laruelle et al., 1994
^11^C-Raclopride	D2/3 receptors	2.19 ± 0.18 (*n* = 40)	Berry et al., 2018
^123^I-IBZM	D2/3 receptors	0.86 ± 0.11 (*n* = 10)	Meyer et al., 2008
^11^C-DASB	Serotonin transporters	2.68 ± 0.68 (*n* = 5)	Ginovart et al., 2001
^123^I-ADAM	Serotonin transporters	1.62 ± 0.57 (*n* = 7)	Yang et al., 2008
^11^C-PK11195	TSPO	1.60 ± 0.40 (*n* = 13)	Kropholler et al., 2005
^11^C-PIB	β-amyloid plaques	0.11 ± 0.15 (*n* = 13)	Tolboom et al., 2009
^18^F-FDDNP	β-amyloid plaques	0.05 ± 0.03 (*n* = 13)	Tolboom et al., 2009

*BP_ND_ reduces as a function of target density and radiotracer affinity (e.g., dopamine receptor, serotonin transporter and β-amyloid plaques ligands). The results also depend on method of quantification used, i.e., kinetic modeling with or without input function (details in sections below). Legend: α4β2-nACh receptor = alpha-4 beta-2 nicotinic acetylcholine receptor; mACh receptors = muscarinic acetylcholine receptors; D2 receptors = dopamine type 2 and 3 receptors; TSPO = 18 kDa translocator protein.*

**FIGURE 2:**
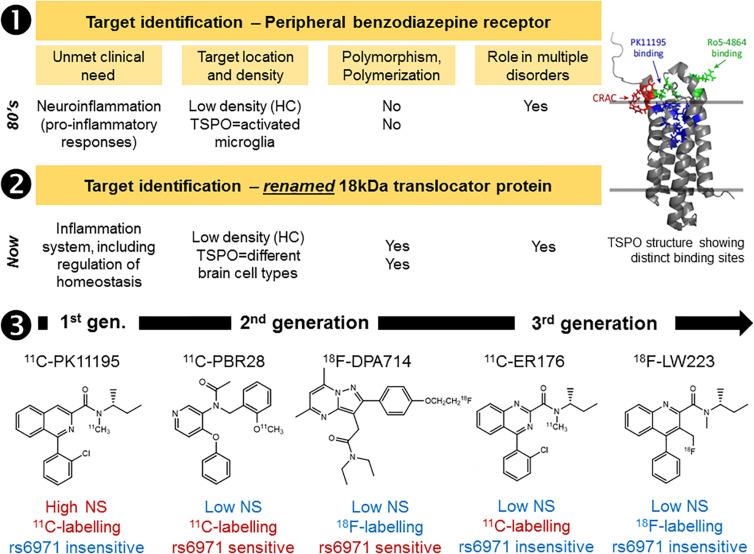
Case study 1: Imaging the 18kDa translocator protein (TSPO), a “moving target” over the past three decades. The development of novel TSPO radiotracers suffered from high degree of attrition over the years (1–3). First there was the renaming of the target, in recognition of the wider functions of this protein. Then the identification of a genetic polymorphism (rs6971) capable of impacting on radiotracer binding in humans (2nd generation, 2nd gen., radiotracers). Alongside these discoveries came the recognition that TSPO has at least three binding sites and can be expressed in a monomer or a polymer configuration depending on the organ or disease process under investigation. Furthermore, limited characterization of the TSPO expression in various cell types decades ago has created a delayed realization that TSPO PET/SPECT imaging was always directed toward understanding TSPO molecular changes rather than cell changes. All these serendipitous findings at the first stage of the radiotracer development pipeline (target identification) have impacted on the development of novel and successful TSPO radiotracers (1 and 2). Despite these difficulties, through a series of compound libraries from 1st generation (1st gen.) radiotracers, like ^11^C-PK11195, to 3rd generation (3rd gen.) radiotracers like ^11^C-ER176 and ^18^F-LW223, it was possible to resolve the target identification issue, while improving radiotracer properties, such as reduction of non-specific binding (NS) and convenient labeling with fluorine-18 (3). Red and blue text indicates limitations and advantages of each example radiotracer displayed in row 3. HC, healthy control brain. TSPO structure taken from Selvaraj and Stocco, Trends in Endocrinology and Metabolism, 2015, 26(7):341.

Although the majority of brain radiotracers developed to date are small molecules that interact with transmembrane receptors and transporters, other targets are amenable for *in vivo* imaging using PET and SPECT. Some radiotracers can serve as subtracts for enzymes (a prominent example is 2-deoxy-2-[^18^F]fluoroglucose) (Van de Bittner et al., 2014) or can bind to protein aggregates, most notably amyloid and tau proteins (Agdeppa and Spiker, 2009; Nordberg, 2014). Furthermore, radiotracers targeting nuclear receptors have also been investigated. These require penetration of the cell nucleus by the radiotracer (Wang et al., 2014). Radiotracers designed to bind to a transmembrane protein, including G-protein coupled receptors, may have to compete with native ligands or allosteric modulation. This means that variations in the endogenous ligand concentrations can impact image quantification of receptor density *in vivo;* setting strict requirements for the candidate radiotracer *K*_*D*_ value. Furthermore, transmembrane receptors have regulatory mechanisms, such as homo- or heteropolymerization and internalization (Ferré et al., 2014); these can represent unexplored avenues in the development of new therapies (George et al., 2002), but directly impact the radiotracer kinetics and binding affinities *in vivo*. Some radiotracers developed to image the 18-kDa translocator protein (TSPO) are also influenced by a genetic polymorphism that leads to differences in binding affinity within the population (Owen et al., 2012). If a novel target has a known potential for genetic polymorphisms, it is important to consider potential effects of genetic polymorphism on radiotracer binding, and thus imaging. Although, interestingly, the TSPO radiotracer ^11^C-PK11195 binding does not seem to be affected by the genetic polymorphism (Owen et al., 2010; Owen et al., 2011). This recent observation of genetic polymorphisms impacting radiotracer binding highlights another new feature to consider when selecting the target for radiotracer discovery (Figure 2, Case study 1).

Another important consideration while discovering novel radiotracers is to understand the error associated with the imaging technique to be use. Typically for any PET and SPECT radiotracers this is represented by the intra-subject test-retest variability. The majority of brain radiotracers have test-retest results of less than 15% [see for example (Abi-Dargham et al., 1995; Seibyl et al., 1996; Booij et al., 1998; Varrone et al., 2000; Catafau et al., 2005; Laere et al., 2013; Barret et al., 2014b; Tavares A. et al., 2014)], meaning that when investigating longitudinal changes in either target density or receptor occupancy in a single-subject, observed changes must exceed 15% in order to be successfully imaged. Various outcome measures are used to quantify test-rest variability. *BP*_*ND*_ is a preferred outcome measure but is not always possible to measure as it requires dynamic imaging data from radiotracers that have reversible binding kinetics and, depending on the target of interest, can also require invasive arterial blood collection for kinetic modeling analysis when a reference region devoid of binding is not available (Innis et al., 2007). *BP*_*ND*_ from distribution volume ratio (*DVR*), calculated from *V*_*T*_, also requires arterial input function data. The total volume of distribution (*V*_*T*_) has also been used for quantification of test-retest variability and as an outcome measure in some cases, such as, in the measurement TSPO radiotracers concentration in the CNS (Sandiego et al., 2015; De Picker et al., 2019). *V*_*T*_ also requires dynamic imaging protocols and the use of arterial input function or, even possible, image derived input function. Standard Uptake Value Ratio (SUVr) is also used in some cases such as for amyloid radiotracers (Knešaurek et al., 2018). For estimation of SUVr, static imaging protocols are sufficient.

Despite all the considerations given during target identification, the most practical and commonly used approach in finding potential radiotracers is to identify high-affinity ligands with known structure-activity relationships. These are often discovered through drug development programs; although highly novel targets without known ligands can also be pursued in radiotracer discovery. Highly novel targets without known ligands can, however, be time and cost-intensive, as various compound libraries need to be developed and screened iteratively. Such “risky” targets, therefore, must have a high *B*_*max*_ and demonstrate a significant percentage change in density or occupancy relating to the research or clinical question being addressed, in order to be worth the input of radiotracer development.

In summary, it is important to investigate which target(s) play an important role in a given disease and can also be imaged using PET or SPECT (considering, for example, the test-retest variability of the technique). Targets that play important roles in multiple disorders can broaden the range of applications of the novel radiotracer candidate thus make more attractive targets. Suitable target density for high quality *in vivo* imaging should be evaluated and the researcher should also pinpoint the desirable affinity of the novel candidate radiotracer at this stage (Figure 1).

### Design and Selection of Lead Radiotracer Candidate

It is tempting to start the process of radiotracer discovery by targeting existing therapeutic drugs, however, it is important to be aware of the negative aspects associated with this choice. For example, therapeutic drugs aim for a sustained target-engagement after one single administration, but this can be unsuitable for radiotracer imaging; resulting in radiotracers with slow kinetics. ^18^F-F13714 is an example of a brain radiotracer with slow kinetics. It was developed as a serotonin type 1A (5-HT1A) receptor agonist radiotracer, however, it was found to have such high affinity that its binding resulted in quasi-irreversible kinetics (Tavares et al., 2013; Yokoyama et al., 2016). This sustained binding maybe useful for drugs, however, is sub-optimal for a radiotracer, as it makes quantification difficult using traditional kinetic models that assume reversible Michaelis-Menten kinetics (Innis et al., 2007). Furthermore, it is important to consider the fact that radiotracers require high brain/plasma ratio and should have low non-specific binding, two factors that can at times be less pressing for therapeutic drugs, where it is not infrequent to have a drug able to bind to several targets in the brain. An example of a non-selective radiotracer is the SPECT iodinated analog of Siponimod (a licensed drug for treatment of Multiple Sclerosis), ^123^I-MS565, which binds to two types of sphingosine-1-phosphate receptors (S1P1 and S1P5) (Tavares A. et al., 2014). It is useful for the use in studying the effects of Siponimod, however, is of limited use when trying to label and track changes in one receptor type in the CNS or to try and establish the role of one of the receptor types in disease development and progression.

**FIGURE 3:**
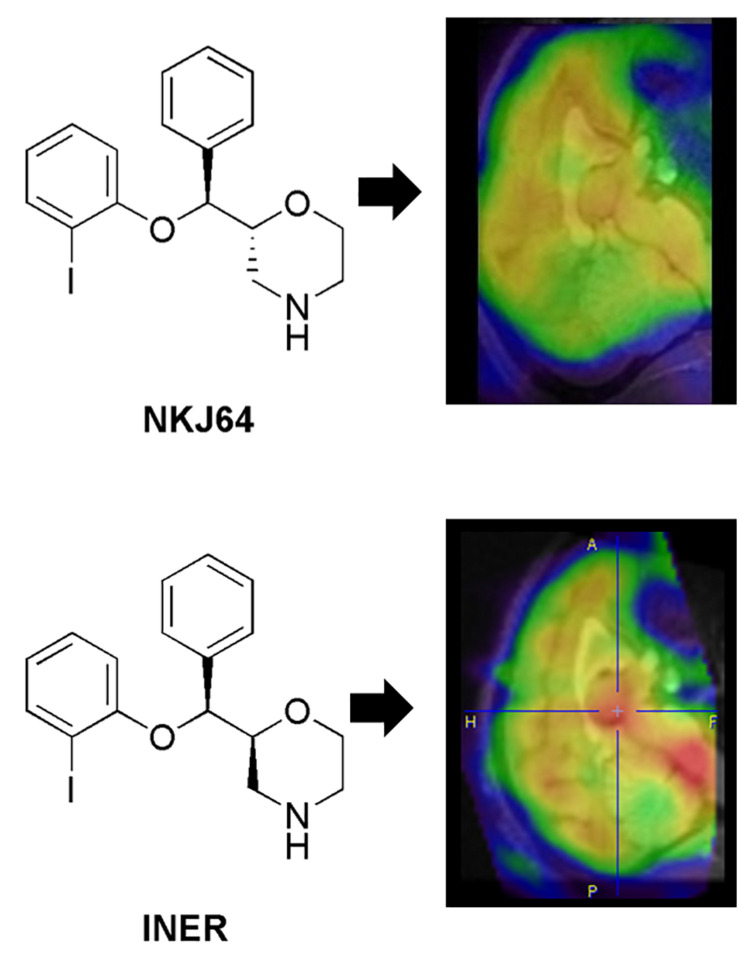
Case study 2: Analog but different. This case study illustrates the impact of small changes in radiotracer structure on binding kinetics *in vivo* and subsequent impact on the translational potential of radiotracers. NKJ64 and INER were both ^123^I-labeled tracers targeting the noradrenaline transporters (NAT). Both stereoisomers had nanomolar affinity *in vitro*, but different kinetics *in vivo*. The INER compound presented binding in brain consistent with NAT expression (highest in the midbrain, in particular locus coeruleus – in red), while NKJ64 had no specific binding to target in baboon brain. SPECT images co-registered to MRI data for the same baboon. A, anterior; P, posterior; F, feet; H, head.

*De novo* radiotracer synthesis is another route for lead candidate selection during radiotracer discovery. This route can be even more iterative than the approach described in the above paragraph. This is particularly true for novel radiotracers that target complex mechanisms or use non-traditional (non-small) compounds, as *in vitro* screening methods would be more limited in predicting their *in vivo* performance. In these cases, conducting simple animal model studies early could provide information and help to optimize the novel radiotracer candidates’ performance *in vivo*. Feedback from preclinical scientists to the chemists can be used to improve the radiotracer’s structure before moving into more rigorous preclinical studies, and if successful, clinical studies (Agdeppa and Spiker, 2009). Another approach to improve the likelihood of discovering high-affinity, brain penetrant molecules during *de novo* synthesis of CNS radiotracers includes the concurrent synthesis of compounds with different chemical scaffolds, as well as, scaffolds that offer several sites for easy substitution with various functional groups. Furthermore, when developing compound libraries it is important that the synthesized molecules have relatively minor deviations in chemical structure, as additions of small chemical groups can significantly affect the radiotracer *in vivo* kinetics and binding properties [see for example, Stevenson et al., 2010; Seo et al., 2014 and Figure 3, Case Study 2).

Regardless of the approach used to start the novel radiotracer design process, at this stage, it is important to consider the development of compounds with a facile labeling site with the potential to be radiolabeled across multiple derivatives (Van de Bittner et al., 2014). Using the same labeling site across various derivatives would allow for minor changes in the radiolabeling method between molecules, saving time and money while developing novel radiotracers. The radionuclides to be used for radiolabeling should also be considered during radiotracer design. For PET imaging, the use of carbon-11 (^11^C), nitrogen-13 (^13^N) and oxygen-15 (^15^O), which are isotopes of endogenous elements, allows for radiolabeling of target molecules without changing their chemical properties with ^15^O-Water, ^15^O-oxygen and ^15^O-CO as the examples for ^15^O. Conversely, fluorine is not ubiquitous in endogenous biomolecules, may lead to differences in radiotracer *in vivo* performance from derivative biomolecules. This is exemplified by, ^11^C-DOPA and *L*-6-fluoro-^18^F-FOPA having different decarboxylation rates *in vivo* (Torstenson et al., 1999). *L*-6-fluoro-^18^F-FOPA can be successfully used for PET imaging, however, it is far more heavily metabolized than its carbon-11 counterpart and requires the use of inhibitors (carbidopa with or without entacapone) to reduce this metabolism and to gain reliable PET images (Kumakura et al., 2005; Kyono et al., 2011; Walker et al., 2013). Importantly, the fluorine-19 (^19^F) commonly present in drug pharmacophores may represent a viable strategy to radiotracer discovery, as in those instances there is only a need for substituting ^19^F in the drug with ^18^F in the radiotracer without changing compound kinetic properties. When the drug pharmacophores do not include ^19^F, one commonly suggested approach involves the use of ^11^C for early probe development, i.e., proof-of-concept *de novo* radiotracer discovery. Once the hypothesized *in vivo* performance using the ^11^C-labeled radiotracer is confirmed, ^18^F-labeled analog or other relatively longer-lived radioisotopes (e.g., iodine-123 for SPECT or iodine-124 for PET) would be explored for subsequent clinical or commercial use. Although this approach is appealing due to the simplicity of substituting elemental carbon with its isotope counterpart ^11^C, from a long term development perspective it adds cost in the process of radiotracer development; with potentially no return and/or will substantially limit the market distribution of the ^11^C-labeled radiotracer, as it is estimated that about 90% of PET scanners are in facilities unable to produce ^11^C radiotracers with high specific activity (Klunk and Mathis, 2008). Still, when investigating highly novel and unexplored targets, this may be the fastest option. It might also be advantageous to use SPECT radiolabels (e.g., ^123^I or Technitium-99m, ^99*m*^Tc) for biomolecules or drug labeling, as there is a long history of labeling proteins, nucleic acids and small molecules with radioiodine (Agdeppa and Spiker, 2009). Other radionuclides may become more prominent in the future as universal procedures for radiolabeling intact monoclonal antibodies (mAbs) or mAbs fragments become available and increasing interest in using these biomolecules in brain imaging is raised within the imaging community (Dongen et al., 2012; McLean et al., 2012). These include the long-lived positron emitters iodine-124 (^124^I, t_1/2_ of 100.3 h) and zirconium-89 (^89^Zr, t_1/2_ of 78.4 h); and the shorter-lived positron emitters gallium-68 (^68^Ga, t_1/2_ of 1.13 h), copper-64 (^64^Cu, t_1/2_ of 12.7 h), yttrium-86 (^86^Y, t_1/2_ of 14.7 h) and bromine-76 (^76^Br, t_1/2_ of 16.2 h). The commercially available long life-span ^68^Ge/^68^Ga-generator (half-life 271 days) makes ^68^Ga a particularly interesting radionuclide for clinical use, as it can be continuously available even for centers without a cyclotron and at relatively reasonable costs.

Another aspect to consider during the design of the radiotracer candidate relates with the development of the precursor for radiolabeling. The precursor should allow for a highly reproducible reaction, automation of the radiosynthesis process (labeling, purification, and formulation) and a radiochemical yield of the formulated product high enough to permit human application (Brust et al., 2014). The automation is useful in various ways, in terms of reproducibility, it minimizes human error. Importantly, once the radiosynthesis method is optimized and is fully automated, production of radiotracers can be scaled-up enabling large scale production for local use (multiple patients a day) or distribution to different PET sites (if the radioisotope half-life permits). Several aspects require consideration when designing candidate radiotracers, including type of precursor to be used. For example, tin precursors are sub-optimal for clinical use, because they require additional quality assurance given the highly toxic nature of tin. The type of precursor selected will also impact on the separation method to be used to obtain the final product (e.g., high performance liquid chromatography or solid phase extraction). Two excellent reviews have been recently published detailing PET radiochemistry principles (Brandt et al., 2018; Pichler et al., 2018).

Identifying the lead radiotracer candidate among a library of compounds shares similarities with the process of drug development and includes screening of the affinity for the target, selectivity, metabolism, lipophilicity and molecular weight/size (Agdeppa and Spiker, 2009; Pike, 2009; Van de Bittner et al., 2014) (Table 2). Despite the comprehensive list of characteristics that hold promise to yield an ideal radiotracer for brain imaging, the suggested criteria are not a definite recipe for success, but rather some empirical or observation-based guidelines that are continuously being refined. In fact, several physicochemical properties such as Log P, Log D, molecular weight and the acid dissociation constant (pK_*a*_) have been found to correlate with *in vivo* behavior to some extent, but are not necessarily predictive of radiotracer performance *in vivo* (Van de Bittner et al., 2014). Thus, even though a substantial emphasis has been placed in the *in silico* models and computation for assessment of physicochemical properties of potential radiotracers for brain imaging (Clark, 2003; Agdeppa and Spiker, 2009), these methods do not necessarily concur with the *in vivo* performance of a number of radiotracers developed, as has been shown in the literature (Tavares et al., 2012b; Van de Bittner et al., 2014).

**TABLE 2:** Criteria for passive diffusion of a radiotracer across the blood-brain-barrier (BBB) and good bioavailability (Lipinski et al., 2001; Clark, 2003; Waterhouse, 2003; Wong and Pomper, 2003; Pike, 2009; Van de Bittner et al., 2014).

Action	Characteristic	Requirement
Crossing BBB	Molecular weight	<450 g/mol
Crossing BBB	Polar surface area	<60–90 Å
Crossing BBB	Number of hydrogen bond donors	<5
Crossing BBB	Sum of nitrogen and oxygen atoms	<10
Crossing BBB	Log P (partition coefficient)	<4 (ideal range of 1–3.5)
Crossing BBB	Affinity for efflux pumps (e.g., P-gp)	Minimal
Crossing BBB	Affinity for enzymes at the BBB	Minimal
Crossing BBB	Brain uptake (%ID)	≥0.5%
	Brain uptake (SUV)	>2.0
Metabolism	Ionization at physiological pH	Low
Metabolism	Presence of radiometabolites in brain	Low
Binding to silent sites	Binding to plasma proteins	Low
Binding to silent sites	Non-specific or non-saturable sites	Low
Binding to target site	Kinetics quantifiable *in vivo*	Reversible or not completely irreversible
Binding to target site	Dissociation or inhibition constants	Nanomolar range
Binding to target site	Selectivity	High
Binding to target site	Specificity	High
Radiological safety	Whole-body and critical organ dosimetry	Low radiation dose

*P-gp, P-glycoprotein; %ID, % injected dose; SUV, standard uptake value.*

Radiotracer metabolism (discussed further in following section) is often a major stumbling point encountered during radiotracer discovery and translational imaging. Although radiotracer metabolism *in vivo* may be hinted during radiotracer design or radiotracer screening *in vitro*, metabolism is highly species-dependent; typically compounds show more extensive metabolism in lower organisms.

**FIGURE 4:**
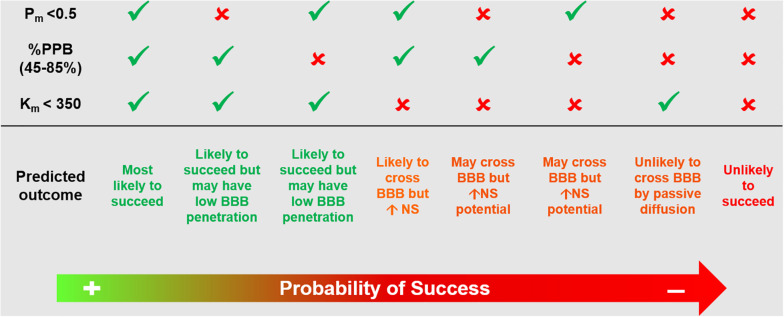
Example of high performance liquid chromatography approach that could be used for screening and ranking of radiotracer candidates. Based on previously published methodology (Tavares et al., 2012b). This chromatography method uses compound retention time information to derive *P*_*m*_, %PPB and *K*_*m*_. These chromatography outcome measures can then be used to predict *in vivo* behavior of the compound using mathematical equations describing the relationships between chromatography outcome measures and *in vivo* measures (%injected dose in brain and binding potential *BP*_*ND*_). *P*_*m*_, permeability; %PPB, %plasma protein binding to human serum albumin; *K*_*m*_, compound-membrane partition coefficient; BBB, blood brain barrier; NS, non-specific binding.

Several methods have been proposed to assist with radiotracer lead candidate selection early in the process of radiotracer discovery. These aim to increase the likelihood of success of a novel brain radiotracer *in vivo*. These methods include *in silico* simulations, *in vitro* testing, high-performance liquid chromatography methods and liquid chromatography–mass spectrometry techniques (Clark, 2003; Agdeppa and Spiker, 2009; Tavares et al., 2012b; Barth and Need, 2014; Van de Bittner et al., 2014). Nonetheless, there is a growing indication from the radiotracer development community that progressing rapidly into *in vivo* imaging following a short high throughput screening exercise targeting key properties (affinity, selectivity and BBB penetration estimation) or even move directly into radiolabeling and *in vivo* imaging studies, with subsequent iterative feedback between preclinical imaging and chemistry, may be the way forward. In this emerging philosophy, the lead candidate selection can be reduced to at most a rapid screening and ranking of molecules prior to radiolabeling, where the affinity for the target would be assessed in conjunction with the biological target density and the likelihood of BBB penetration would be assessed by a high throughput technique. To maximize efficiency, during radiotracer affinity experiments, it is useful to include a known radiotracer targeting the same process as a control together with the radiotracer library under scrutiny. This has already been applied in the development of novel TSPO ligands based on PK11195 structure (Stevenson et al., 2010) (Figure 2, Case Study 1). Although the specific ranking order of the compound’s affinity is less central than their clustering based on chemical structure, it can provide a starting point to select which cluster of radiotracer candidates to prioritize for *in vivo* imaging. Similarly, when ranking candidate radiotracers for BBB penetration, it is important to include in the analysis known successful radiotracers. A recently proposed methodology uses high performance liquid chromatography for rapid screening and ranking of candidate radiotracers in terms of permeability, plasma protein binding and compound-membrane interaction (Tavares et al., 2012b) (Figure 4) and has been adopted by different research groups working on novel radiotracer development (Blair et al., 2013; Gilfillan et al., 2013; Mark et al., 2013; Philippe et al., 2013a, b; Rami-Mark et al., 2013). This chromatography method uses compound retention time information to derive permeability, plasma protein binding and compound-membrane interaction and vs. a calibration curve. The chromatography outcome measures (permeability, plasma protein binding and compound-membrane interaction) can then be used to predict *in vivo* behavior of the compound using mathematical equations describing the relationships between chromatography outcome measures and *in vivo* measures (%injected dose in brain and *BP*_*ND*_) (Tavares et al., 2012b). Newer mass spectrometry methods have also been proposed for estimation of specific and non-specific binding across brain regions (Barth and Need, 2014). However, the use of unlabeled compounds makes the measurement of metabolites difficult and the proposed approach can be labour intensive. It requires the use of several animals and dissection techniques following administration of the unlabeled compound, as each animal can only provide information for a limited number of time points.

In summary, the design and selection of the lead radiotracer candidate can start by targeting existent therapeutic drugs or initiating a *de novo* radiotracer program. During this stage, the researcher should focus on targeting compounds that would allow for ease radiolabeling, preferably would allow for labeling with different radioisotopes for PET or SPECT imaging (keeping the potential market options open). Once the library of target compounds has been identified or designed, screening of these compounds for key properties (affinity and BBB penetration) must be rapid and simple, with the aim to progress as quickly as possible to *in vivo* imaging. Sometimes it might even be possible to obviate this step all together and move straight into radiolabeling and *in vivo* imaging, depending on the level of information one has on a given molecule (Figures 1, 4).

### Preclinical Studies to Evaluate Novel Radiotracer Candidates

For decades, the affinity, selectivity and binding kinetics of novel radiotracers have been studied using autoradiography and binding assay techniques (Udenfriend et al., 1987; Cook, 1996; Schuck, 1997; Qume, 1999; Lazareno, 2001; Frank et al., 2007). However, frequently, these approaches lead to novel radiotracers displaying poor *in vivo* kinetics and high non-specific binding in the first round of *in vivo* imaging, often contributing to the high attrition rate during radiotracer development. Thus, moving the novel radiotracer rapidly into small animal *in vivo* imaging can accelerate the process of radiotracer discovery and development (de Kemp et al., 2010; Koba et al., 2013) by removing redundant steps between compound chemical synthesis and *in vivo* imaging. A successful example that came out of this direct strategy, supported by *in vivo* imaging at early stages, is ^18^F-MNI444, a novel PET radiotracer targeting adenosine-2A receptors (A2A) in the brain (Barret et al., 2014a; Barret et al., 2015). Preladenant served as target re-purposed structure for development of this radiotracer, then precursors for radiolabeling were designed and synthesized and the new radiotracer immediately tested in non-human primates (Barret et al., 2014a). The promising results in non-human primates rapidly prompted the progression of the compound to humans (Barret et al., 2015). The whole process took only 4 years.

In fact, imaging studies can allow for the determination of major radiotracer attributes traditionally determined using non-imaging methods or *in vitro*/*ex vivo* imaging techniques. For example, *in vivo* imaging can be used to determine binding affinity (BP = *B*_*max*_/*K*_*D*_, where smaller *K*_*D*_ would corresponds to larger BP for a given target); binding kinetics can be investigated by quantitative analysis and kinetic modeling of the obtained time-activity curves; BBB penetration can be estimated as percentage injected dose in brain; specific binding can be determined using homologous blocking experiments or knockout animals; and selective binding can be determined using heterologous blocking or knockout animals (Kemp et al., 2010; Koba et al., 2013; Van de Bittner et al., 2014). Nowadays, kinetic modeling in rodents much as is done in humans and large animals is becoming more commonplace due to advances in automatic blood sampling instrumentation (Alf et al., 2013; MacAskill et al., 2019). This, however, does have some limitations, namely the fact that it requires expertise in femoral artery cannulation, a procedure that can only typically be performed twice in small animals (Warnock et al., 2014). For those PET/SPECT research sites without access to this blood sampling technology, manual blood collection is possible and would enable the use of population-based curves, or image-derived input functions can be obtained. Although these would result in quantitative bias vs. the continuous arterial sampling per experiment (MacAskill et al., 2019), they can still allow the researcher to quantify PET data and conclude on which radiotracer candidate is the most promising for translation to higher species. Furthermore, moving a novel radiotracer from chemistry directly into *in vivo* preclinical imaging can also reduce the likelihood of researchers to overlook useful radiotracers. For example, the use of autoradiography techniques for assessment of binding kinetics by varying pre-incubation and washing steps would be unsuitable in evaluating whole-body biodidistribution kinetics and metabolism, essential aspects for assuring the success of a novel radiotracer for *in vivo* imaging. Similarly, even the “no wash” autoradiography method proposed for estimation of specific binding (Patel et al., 2003) would be suboptimal at evaluating a novel radiotracer performance *in vivo* (the ultimate targeted environment). Moreover, the resources allocated for these type of *in vitro*/*ex vivo* experiments require radiolabeling of the radiotracer, deeming these approaches inadequate for prospective screening. Consequently, these techniques are slowly, but consistently, being replaced by preclinical imaging techniques, which are becoming key translational tools for proof-of-mechanism and concept studies (Agdeppa and Spiker, 2009; Van de Bittner et al., 2014) (Figure 1). That said, the use of *ex vivo* studies to assess radiotracer metabolism is still unavoidable, given the need to sample organ tissue and blood for analysis (as detailed below).

Another important factor that may explain the reasoning behind using *in vivo* preclinical imaging methods vs. *ex vivo* dissection studies for assessment of radiotracer distribution is that imaging devices are seen as technical refinements, in line with the principles of the three Rs (replacement, reduction and refinement). There are several reasons supporting this view, including the fact that: (1) *in vivo* imaging techniques are less invasive that other techniques; (2) by using the animals as their own controls, the number of animals to be sacrificed is reduced and the statistical power is improved, with consequent scientific benefit; and (3) diagnostic and therapeutic agents can be developed on identical platforms, thus providing a unique straightforward translational paradigm. In fact, small animal imaging has been perceived as an approach with potential to provide a natural bridge to the clinical development by the National Institute of Biomedical Imaging and Bioengineering established by the National Institutes of Health (Koba et al., 2013).

Preclinical *in vivo* imaging can be used early in radiotracer discovery to make modifications of the chemical structure based on the novel radiotracer kinetic behavior, allowing for a faster “tailoring” of the most suitable radiotracer candidate. As discussed throughout this review, moving toward this approach could enable rapid and successful translation of novel brain radiotracers to clinical use. Furthermore, preclinical imaging can alternatively be used later (prior to human studies) to obtain the complete kinetics of the radiotracer, as well as, to estimate dosimetry, in order to identify the dose-limiting organ radiation exposure; information that can be used in the translation to humans. In fact, the recently developed MOBY mice phantoms for estimation of radiation exposure using mice models have made it possible to use preclinical imaging for dosimetry estimates, easing translation into humans (Larsson et al., 2007). Although *ex vivo* biodistribution studies can also be used to estimate dosimetry, they require at least 4-6 times more animals, given that each measurement at a given time point requires sacrificing a group of rodents for tissue harvesting, vs. dynamic *in vivo* imaging of multiple points from a single rodent. The use of preclinical dosimetry for estimation of clinical radiological safety has been a regulatory requirement for several years, albeit some recent discussion has questioned its utility given the first in human studies include dosimetry assessments. It is possible that the development of highly sensitive scanners, e.g., the total-body PET (Cherry et al., 2018), would enable even faster translation to humans by minimizing concerns around radiation dose exposure from novel radiotracers.

Given that PET or SPECT radiotracers should not elicit a pharmacologic effect (owing to the radiotracer principle), a pharmacodynamics response is not measurable. This means the burden of proof lies in the ability to correlate the uptake with modulated target density. This can be easily accomplished using *in vivo* imaging techniques and therapeutics or genetically modified animals (Agdeppa and Spiker, 2009). Assessment of radiotracer brain penetration can be accomplished by inspecting the time-activity curves and expressing the measured signal as a percentage injected dose over time. The cutoff values for accepting a radiotracer as suitable for CNS imaging based on whole-brain uptake are not consensual. Recently, Van de Bittner et al. (2014) reported the values used in their laboratory to be 0.1% injected dose (ID)/cc for rats and 0.01% ID/cc for non-human primates within 5 min of injection. As mentioned previously, values of brain uptake in rodent and non-human primate of ≥ 0.5% have also been suggested as a guideline to yield suitable CNS radiotracers. However history has shown that, in humans, BBB-penetrant radiotracers developed for *in vivo* imaging of the CNS have expressed variable%ID in whole-brain. For example, ^123^I-PK1119, ^11^C-DASB and ^123^I-Iomazenil had a peak %ID in whole-brain of around 2.0% (Versijpt et al., 2000), 4.0% (Lu et al., 2004), and 13% (Dey et al., 1994), respectively. This represents a fairly wide range of radiotracer brain uptake, highlighting that definitive cutoff limits might be difficult to implement. This is because %ID values provide little information about radiotracer kinetic properties and are dependent on a number of factors, including radiotracer free fraction in plasma and time of measurement after injection. Still, cutoffs proposed in the literature, as a result of different groups experience while developing radiotracers, can be a useful first line guidance when screening compounds (e.g., by using *in vitro* methodology reported in Figure 4 and in Tavares et al. (2012b). Another method proposed to evaluate radiotracer penetration is based on the analysis of the standard uptake values (SUVs). This has the advantage of allowing for comparison across different species and tissues, as it is a measure normalize to injected dose and body weight. An SUV value of 1 will correspond to a radiotracer concentration that would result in uniform distribution of the injected dose in the whole-body. Typically, a peak SUV > 2 is generally desirable for brain imaging (Pike, 2009) (Table 2).

Subsequent to demonstrating radiotracer brain penetration, it is important to assess the degree and length of radiotracer retention in the brain. For example, evaluating if the radiotracer has been actively effluxed from the brain. This can be accomplished by imaging animals pretreated with inhibitors of active efflux proteins. Active efflux mechanisms show variation across different species and, similarly to metabolism, can be difficult to control or predict when translating a radiotracer from one species to another. In a study comparing three PET radiotracers in different species, it was found pronounced differences in the brain and brain-plasma concentrations of ^11^C-Verapamil, ^11^C-GR205171 and ^18^F-altanserin with lower brain distribution in rats and guinea pigs compared with humans, monkeys and minipigs. One of the conclusions of that study was that compounds found to be P-gp substrates in rodents are also likely to be substrates in higher species, although this is not a linear predictor of BBB permeability and the compound might still be retained in human brain (Syvänen et al., 2009). In general, the majority of the P-gp substrates are transported to a lower extent by human P-gp compared with mouse or rat P-gp (Yamazaki et al., 2001; Ohe et al., 2003; Katoh et al., 2006; Baltes et al., 2007). However, there are exceptions, for example, cyclosporin A (Katoh et al., 2006; Baltes et al., 2007).

When a radiotracer candidate displays good brain penetration and retention, specific binding to the target can be demonstrated by homologous blocking studies. During these measurements it is important to consider potential blockade of the radiotracer binding in peripheral tissues, resulting in increased free radiotracer in plasma with consequent increase of total brain uptake relative to control. This effect can be corrected by normalizing the radiotracer uptake to metabolite-corrected plasma radiotracer levels or by performing kinetic modeling analysis of the acquired data. Furthermore, heterologous blocking studies can be used to evaluate potential off-target binding and knockout animals can be of value for confirming on-target binding.

Analyzing the time-activity curves from *in vivo* preclinical imaging experiments, can infer the suitability of a radiotracer for human imaging. For example, if the measured time-activity curve slope is near zero, this may be indicative of irreversible binding or very slow kinetics, which is not ideal *in vivo*. Conversely, decreases in binding potential during bolus or bolus plus constant infusion experiments with injection of homologous or heterologous blocking agents midscan represent measurable adequate reversible kinetics (Van de Bittner et al., 2014).

Effective radiotracer design can limit potential problems from troublesome radiometabolites, yet radiotracers resistant to extensive metabolism over the duration of the PET or SPECT imaging session are scarce (Pike, 2009). And as mentioned before, the nature and degree of metabolism varies across species. For example, the metabotropic glutamate receptor 5 (mGluR5) radiotracer ^18^F-SP203 (Siméon et al., 2007) is rapidly defluorinated in the rat brain and blood as well as in monkey blood, however, shows no defluorination in humans (Brown et al., 2008). A similar trend has been observed with the serotonin type 4 receptors (5HT4) radiotracer, ^11^C-SB207145, that was successfully translated into humans (Gee et al., 2008; Marner et al., 2009), yet displayed pronounced peripheral metabolism in preclinical species. Therefore, it has been argued that the determination of metabolites in rodents or larger animals (pigs or monkeys) provides suitable data to inform clinical PET studies. The argument is that due to the higher surface-to-volume ratio, the influence of metabolism on the PET quantification of human data is usually overestimated when investigated in experimental animals (Brust et al., 2014). Due to the known species differences, it has been argued that efforts should be made to progress to first in human studies as quickly as possible and some have argued that non-human primate preclinical imaging would be more predictable of radiotracer performance in humans (Van de Bittner et al., 2014), although this might not always represent the truth. In fact, for certain complex CNS targets, it might be that small animal models are more suitable to investigate a novel radiotracer, despite the potential for a faster metabolism. Genetically modified rodent models that express human disorder phenotypes are readily available and produce information that may not be so easily obtained from non-human primates. For example, when developing a novel radiotracer targeting neuroinflammation or beta-amyloid deposition, conditions that are not present in a healthy brain, it can be difficult to judge the novel radiotracer candidate performance without the use of adequate animal models. Weighing the pros and cons of using different animal species for radiotracer development demonstrates why radiotracer metabolism can be considered one of the parameters most difficult to control during radiotracer discovery, and the one potentially causing the most attrition during this process.

Ideally the metabolism of a novel radiotracer would occur outside the brain and produce less lipophilic radiometabolites, than the parent radiotracer, with poor brain penetration. However, this is not always the case. Thus, at times, the rate at which troublesome radiometabolites cross the BBB into the brain parenchyma and their affinity for the target determines the performance of a radiotracer. For example, the presence of non-troublesome radiometabolites in the brain has been reported for several novel radiotracers developed to image phosphodiesterases type 10A (PDE10A) (Celen et al., 2010; Tu et al., 2011; Celen et al., 2013; Van Laere et al., 2013; Barret et al., 2014b). These studies demonstrate a region-dependent metabolite fraction profile, with the highest fraction in the cerebellum and lowest in the caudate/putamen (the target region) (Celen et al., 2010; Celen et al., 2013). This suggests that the radiometabolite entering the brain does not bind to PDE10A and only gives non-specific signal. Furthermore, signal in the brain from the radiometabolite seems consistent, low and negligibly reflected in the *in vivo* time-activity curves of regions with low densities of PDE10A. These observations have allowed for the successful translation of the novel PDE10A radiotracers from preclinical into clinical research, even with the presence of radiometabolites in the brain (Barret et al., 2014b).

As stated throughout this review, a multifactorial and complex issue that causes attrition throughout the radiotracer development pipeline is species differences. Several views on this topic have been largely discussed in the literature. Some defend moving the novel brain radiotracer directly into non-human primate without small animal imaging, to minimize the attrition rate when translating to humans. This view is supported by data demonstrating a close homology between, for example, amino acid content in P-gp in humans compared with non-human primates (93% homology between humans and rhesus monkey, vs. only 85% between human and rat) (Syvänen et al., 2009). Furthermore, target density in brain might be significantly different in rat compared with non-human primate and human. For example, in the non-human primate brain, the density of noradrenaline transporters (NAT) in the locus coeruleus is around 220 fmol/mg, while in rodents the NAT density in the same brain region is around 1500 fmol/mg (Tejani-Butt, 1992; Smith et al., 2006). Thus, rat brain has seven times more NAT binding sites than non-human primate brain. This has been pointed out as one of the potential reasons underlying the different imaging profile measured with ^123^I-NKJ64 (a SPECT radiotracer candidate for imaging of NAT in brain) in rats compared to baboons (Tavares et al., 2011; Tavares et al., 2012a). It is worth noting that that the density of NATs in human locus coereleus is similar to the one measure in non-human primates (Ordway et al., 1997). Consequently, although rat data demonstrated that ^123^I-NKJ64 held promise for imaging of NAT in brain, data collected in baboons showed a lack of specific signal in target regions and precluded the translation of this radiotracer into humans (Figure 3, Case Study 2). Nonetheless, there might be instances where the use of rodents may be preferred when developing novel radiotracers. The mouse remains the premier animal model for biomedical research because mice have a short reproductive cycle, can be easily genetically modified and are easy to maintain. Furthermore, there is high homology between the human and the mouse genomes, which facilitates the construction of animal models of human disease at a fast rate and with high specificity (Niu and Chen, 2014). There are multiple transgenic animals containing mutations for human disease for with a specific gene has been identified, including for example, Parkinson’s disease, Huntington’s disease, Alzheimer’s disease, epilepsy and multiple sclerosis (Furlan et al., 2009; Casteels et al., 2014). Also, the introduction of the “humanized mouse” models may serve as a preclinical bridge for translating data from animal models into humans (Niu and Chen, 2014). Rodent models may be the best preclinical alternative available when developing novel radiotracer targeting neuroinflammatory human brain disorders, such as multiple sclerosis, or brain disorders that result from abnormal protein accumulation that is not observed in the healthy brain, given that rodent models are reliable and predictive models of human disorders. This does not mean that non-human primate models of brain disorders are not available, however, they are often chemically- or vector-derived models and come at a higher cost, which may add to an already expensive development process. That being said, the use of small animals in psychiatry is limited, especially when investigating pathways without homology in rodents, e.g., the expanded prefrontal cortex of the human brain (Casteels et al., 2014). Furthermore, the metabolism in lower species such as rodents may not directly forecast metabolism in humans, as shown on various publications, because it tends to be faster in rodents compared with humans. Species differences are unavoidable, however, as outline above, preclinical radiometabolism studies can still provide useful information during radiotracer development, because preclinical studies enable organ collection for direct quantification of radiometabolism in tissue – something that is not feasible in humans. Importantly, preclinical rodent studies often present the “worst case scenario” due to rapid metabolism of these species and can enable comparative analysis of analog in a compound library prior to translation to humans.

In addition to the pitfalls and challenges highlighted above, the majority of the preclinical *in vivo* imaging studies are conducted under anesthesia. The list of publications demonstrating the impact of anesthetic agents on radiotracer uptake and kinetics is vast [see for example: (Lee et al., 2005; Fueger et al., 2006; Hildebrandt et al., 2008; Giron, 2009)], so this will not be discussed in detail here, but it is worth to point out the need to be aware of this potential confounder when interpreting *in vivo* preclinical imaging data. Another important aspect that has also been extensively reviewed is the need to accurately control the levels of administered radiotracer mass. If the radiotracer principle is key to PET or SPECT imaging in human subjects, in preclinical imaging it is imperative that radiotracer levels are controlled owing to the smaller animal body sizes. An example would be the setoronin type 4 receptor (5-HT4) radiotracers, ^11^C-SB207145 and ^18^F-MNI698, where microgram per kilogram mass doses were sufficient to induce non-negligible receptor occupancy (Madsen et al., 2011; Tavares A. et al., 2014). These radiotracers were still useful for preclinical and clinical PET imaging of 5-HT4 because the molar activity was sufficiently high, thus respecting the radiotracer principle. However, these cautionary mass-effect studies highlight the importance of quantifying and respecting the molar activity limits to enable high sensitivity PET imaging. With the recent development of total-body PET human systems (Cherry et al., 2018), the molar activity limits might be less restrictive owing to a gain in instrumentation sensitivity, which would in turn require low injected doses of the radiotracer. Notwithstanding, researchers should always be mindful of the radiotracer principle, especially when developing novel PET or SPECT brain radiotracers.

**FIGURE 5:**
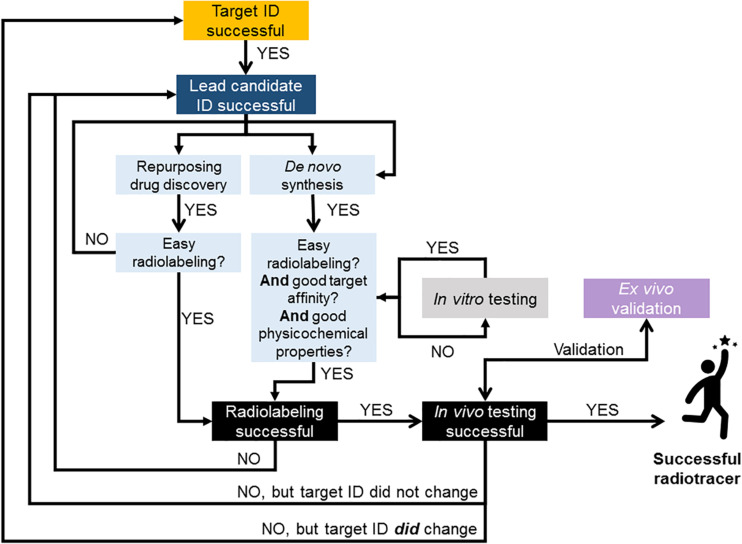
Flow chart illustrating suggested approach to radiotracer discovery and development. Decision points based on literature reviewed in this paper and experience in brain radiotracer development. This illustrates the iterative nature of the process and provides guidance on strategies for successful translation of novel compounds into humans. When possible, repurposing compounds from drug discovery pipelines as potential radiotracer candidates can be advantageous to rapidly and successfully develop new PET and SPECT radiotracers for imaging the human brain. In case of *de novo* chemistry, all *in vitro* testing should be conducted with the non-radioactive library of compounds. This could be done by employing high throughput screening methods for measuring novel compounds’ physicochemical properties (e.g., Figure 4) and affinity for target. The removal of *in vitro* testing of the radiolabeled compound (as per Figure 1) will also further accelerate the process.

In summary, at this stage of radiotracer development, controversies arise when discussing which animal species to use and good arguments from each side of the fence suggest that a case-by-case analysis is necessary when deciding how to progress in the radiotracer development pipeline. The use of non-human primates can provide the most insightful and “predictive” information prior to human studies, however, there are instances when investigating specific CNS targets where the use of genetically engineering rodent models can be more useful than non-human primates in assessing radiotracer performance. There is, however, a growing consensual view that moving straight to *in vivo* preclinical imaging and skipping *in vitro*/*ex vivo* testing with the radiolabeled compound is the most efficient way to proceed during radiotracer development (Figures 1, 5). Importantly, the process of developing radiotracers is iterative and failure at some stage can lead back to an earlier stage, as illustrated in Figure 5.

### Proof-of-Concept in Humans

Once a novel radiotracer has passed through preclinical testing and shows promising results, it will enter the final step in radiotracer discovery/development, i.e., proof-of-concept in humans with subsequent clinical trials. To successfully obtain permission to move a radiotracer from preclinical to clinical use it is important to transition from a research-grade radiochemical to a radiopharmaceutical for which higher standards of product quality must be met (Brust et al., 2014). The list of aspects to consider during this translational process is long and has been extensively reviewed elsewhere (Verbruggen et al., 2008; Agdeppa and Spiker, 2009; Elsinga et al., 2010; VanBrocklin, 2010). Radiation safety, toxicology issues, quality control, licensing and regulatory control are some of the aspects to consider. The regulatory control is often the major bottleneck in the translation process from preclinical to clinical research and has become increasingly restrictive over the last two decades. For example, currently the typical time between the successful radiolabeling of a novel radiotracer and first human use can vary between 5 and 10 years, while at the beginning of neuroreceptor imaging with PET or SPECT this transition was much shorter, in the range of 1–2 years (Brust et al., 2014). Typically the factors contributing to the limited number of approved radiotracers can be classified in three major categories: radiotracer discovery and development, regulatory aspects and clinical development (VanBrocklin, 2010). Another hurdle in transitioning from animal research to human studies relies on the fact that the radiotracer may fail and be deemed unsuitable for *in vivo* imaging of the human brain. However, even if the radiotracer is not further developed into a radiopharmaceutical, it may still find widespread use in preclinical studies to investigate animal models of diseases or new drugs. Thus, one should remain optimistic when discovering and developing novel radiotracers for brain imaging, as with persistence and ever-improving preclinical assessment of radiotracers, the translation gap between animal and human research is rapidly tightening.

### Proposed Approach for Rapid Translation of Radiotracers From Animal to Human Research

Developing novel radiotracers for brain imaging is a challenging process, requiring multidisciplinary teams, in order to gather the knowledge from marketing and target identification, disease-related biological mechanisms, chemical synthesis, radiolabeling, and image acquisition and analysis. This process can be lengthy and costly, and circumventing failure is difficult when translating from animal research into clinical use. Several approaches and philosophies have been suggested for improving efficiency of the radiotracer discovery and development pipeline (Nunn, 2007; Salvadori, 2008; Agdeppa and Spiker, 2009; Van de Bittner et al., 2014). An alternative proposed based on the literature reviewed in this paper as well as on current trends and previous experience while developing novel radiotracers is presented as a flow chart in Figure 5.

## Concluding Remarks

Biomarkers can be classified into three classes: type 0 biomarkers along the continuum of the natural history of the disorder; type 1 biomarkers for detecting therapeutic drug’s mechanisms of action; and type 2 biomarkers that are equivalent to surrogate end points (Frank and Hargreaves, 2003). PET and SPECT imaging target all three types of biomarkers. Hence the potential of these imaging modalities is phenomenal alongside concept of personalized medicine, as well as and the key supportive role they can have in the process of novel therapeutics discovery and development. The development of novel radiotracers and subsequent rapid and successful translation into humans is pivotal to realize the potential of PET and SPECT imaging as useful biomarkers. This translational process can be optimized and efficiently shorten with the aim to move into clinical use as soon as possible. A suggested approach to assist with these goals has been proposed in this manuscript, essential aspects to consider when developing novel radiotracers for brain imaging were highlighted and proposed solutions or alternatives, when available, were described.

## Author Contributions

RS, GT, and AT made substantial contributions to the manuscript construction, participated in the drafting of the manuscript, approved the final version of the manuscript, and agreed to be accountable for all aspects of this work. All authors contributed to the article and approved the submitted version.

## Conflict of Interest

GT is the CEO of XingImaging, LLC, United States. The remaining authors declare that the research was conducted in the absence of any commercial or financial relationships that could be construed as a potential conflict of interest.
